# Engineering mutually orthogonal PylRS/tRNA pairs for dual encoding of functional histidine analogues

**DOI:** 10.1002/pro.4640

**Published:** 2023-05-01

**Authors:** Christopher J. Taylor, Florence J. Hardy, Ashleigh J. Burke, Riley M. Bednar, Ryan A. Mehl, Anthony P. Green, Sarah L. Lovelock

**Affiliations:** ^1^ Manchester Institute of Biotechnology, School of Chemistry, University of Manchester Manchester UK; ^2^ Department of Biochemistry and Biophysics Oregon State University Corvallis Oregon USA

**Keywords:** 3‐methyl‐histidine, 3‐pyridylalanine, dual incorporation, genetic code expansion, histidine analogues

## Abstract

The availability of an expanded genetic code opens exciting new opportunities in enzyme design and engineering. In this regard histidine analogues have proven particularly versatile, serving as ligands to augment metalloenzyme function and as catalytic nucleophiles in designed enzymes. The ability to genetically encode multiple functional residues could greatly expand the range of chemistry accessible within enzyme active sites. Here, we develop mutually orthogonal translation components to selectively encode two structurally similar histidine analogues. Transplanting known mutations from a promiscuous *Methanosarcina mazei* pyrrolysyl‐tRNA synthetase (*Mm*PylRS^IFGFF^) into a single domain PylRS from *Methanomethylophilus alvus* (*Ma*PylRS^IFGFF^) provided a variant with improved efficiency and specificity for 3‐methyl‐L‐histidine (MeHis) incorporation. The *Ma*PylRS^IFGFF^ clone was further characterized using in vitro biochemical assays and x‐ray crystallography. We subsequently engineered the orthogonal *Mm*PylRS for activity and selectivity for 3‐(3‐pyridyl)‐L‐alanine (3‐Pyr), which was used in combination with *Ma*PylRS^IFGFF^ to produce proteins containing both 3‐Pyr and MeHis. Given the versatile roles played by histidine in enzyme mechanisms, we anticipate that the tools developed within this study will underpin the development of enzymes with new and enhanced functions.

## 1 INTRODUCTION

**FIGURE 1:**
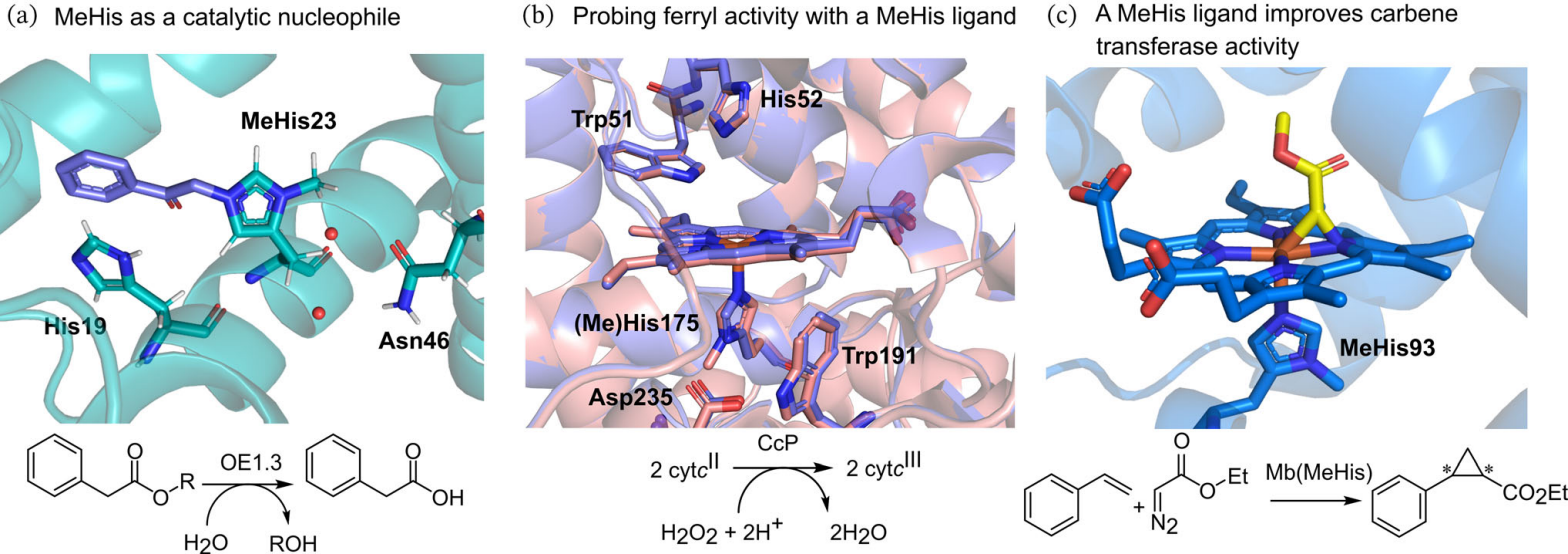
3‐Methyl‐l‐histidine (MeHis) is a versatile residue in enzyme design and engineering. (a) The designed esterase OE1.3 uses MeHis23 as a catalytic nucleophile (Burke et al., 2019). The crystal structure (pdb 6Q7R) shows MeHis23 (atom‐colored sticks, carbons in green) alkylated with the mechanistic inhibitor bromoacetophenone (atom‐colored sticks, carbons in purple). (b) In cytochrome *c* peroxidase, the axial His (atom‐colored sticks, carbons in purple, pdb 1ZBY) to MeHis (atom‐colored sticks, carbons in pink, pdb 6H08) ligand substitution was used to probe the relationship between ligand strength and ferryl reactivity (Ortmayer et al., 2020). (c) Substituting the His93 ligand of myoglobin variants with MeHis (atom‐colored sticks, carbons in blue) affords an oxygen tolerant carbene transferase (Hayashi et al., 2018). The crystal structure (pdb 6G5B) shows the Fe(III) carbenoid complex (atom‐colored sticks, carbons in yellow) generated following reaction with the substrate ethyl diazoacetate.

Enzymes are powerful catalysts capable of promoting reactions with unrivaled efficiencies and selectivities. These favorable properties have inspired efforts to design new catalytic sites in proteins to deliver enzymes for target chemical transformations (Baker, 2010; Hilvert, 2013; Kiss et al., 2013; Lovelock et al., 2022). However, proteins are typically biosynthesized from only 20 canonical amino acid building blocks that contain a limited number of functional groups, which restricts the range of catalytic mechanisms conceivable within designed active sites. The availability of orthogonal aminoacyl tRNA synthetase (aaRS)/tRNA pairs for encoding diverse noncanonical amino acids (ncAAs) into proteins opens new opportunities in enzyme design and engineering (Chin, 2017; Liu & Schultz, 2010). For example, ncAAs have been used as tools to probe complex biological mechanisms (Ortmayer et al., 2020, 2021; Seyedsayamdost et al., 2006; Wu & Boxer, 2006), to enhance enzyme activity and stability (Green et al., 2016; Pott et al., 2018), and to develop enzymes with entirely new catalytic mechanisms (Burke et al., 2019; Drienovská et al., 2018; Mayer et al., 2019; Sun et al., 2022; Trimble et al., 2022). In this way, 3‐methyl‐L‐histidine (MeHis) has proven to be a highly versatile residue in enzyme design and engineering, serving as a catalytic nucleophile in de novo enzymes and as a ligand in metalloenzyme active sites (Figure 1; Burke et al., 2019; Carminati & Fasan, 2019; Hayashi et al., 2018; Pott et al., 2018). As a nucleophile, MeHis operates with a similar mode of action as the widely employed small molecule nucleophilic catalyst 4‐dimethylaminopyridine (DMAP) that can promote a range of valuable chemical transformations (Wurz, 2007). As a metal chelating ligand, MeHis has been used to probe the highly evolved mechanisms of cytochrome *c* peroxidase and ascorbate peroxidase, shedding new light on their complex catalytic mechanisms (Green et al., 2016; Ortmayer et al., 2020). MeHis has also been used to augment metalloenzyme function, for example, its incorporation into the oxygen binding protein myoglobin led to substantial improvements in both peroxidase and carbene transferase activities (Carminati & Fasan, 2019; Hayashi et al., 2018; Pott et al., 2018).

At present, MeHis is most commonly incorporated into target proteins using a promiscuous pyrrolysyl tRNA synthetase (PylRS^IFGFF^) from either *Methanosarcina mazei* (*Mm*) or its homolog *Methanosarcina barkeri* (*Mb*) (Xiao et al., 2014). This PylRS variant is active toward a range of histidine analogues including MeHis and 3‐(3‐pyridyl)‐L‐alanine (3‐Pyr), although elevated ncAA concentrations are required to achieve high suppression efficiency. Here, we show that transplanting the mutations from *Mm/Mb* PylRS^IFGFF^ into the more recently discovered PylRS from *Methanomethylophilus alvus* (*Ma*) not only provided a synthetase (*Ma*PylRS^IFGFF^) with improved MeHis incorporation efficiency, but also led to a more selective enzyme (Willis & Chin, 2018). As *Ma*PylRS is mutually orthogonal to the highly versatile *Mm/Mb* system (Beranek et al., 2019; Dumas et al., 2015; Wan et al., 2014), the availability of *Ma*PylRS^IFGFF^ should allow us to produce proteins containing MeHis in combination with a second functional ncAA (e.g., a second histidine analogue) (Bednar et al., 2021; Wang et al., 2014). As proof of concept, we subsequently engineered a selective *Mm*PylRS variant for encoding 3‐Pyr, and used this system in combination with *Ma*PylRS^IFGFF^ to produce proteins containing both 3‐Pyr and MeHis. Given the large number of enzymes containing multiple functional histidine residues, we anticipate that these methods will provide new opportunities to study enzyme mechanisms and create active sites with enhanced functionality (Krebs et al., 2007; Lewis et al., 2011; Vaillancourt et al., 2006; Walton & Davies, 2016).

## 2 RESULTS

### 2.1 Engineering a selective PylRS for MeHis incorporation

**FIGURE 2:**
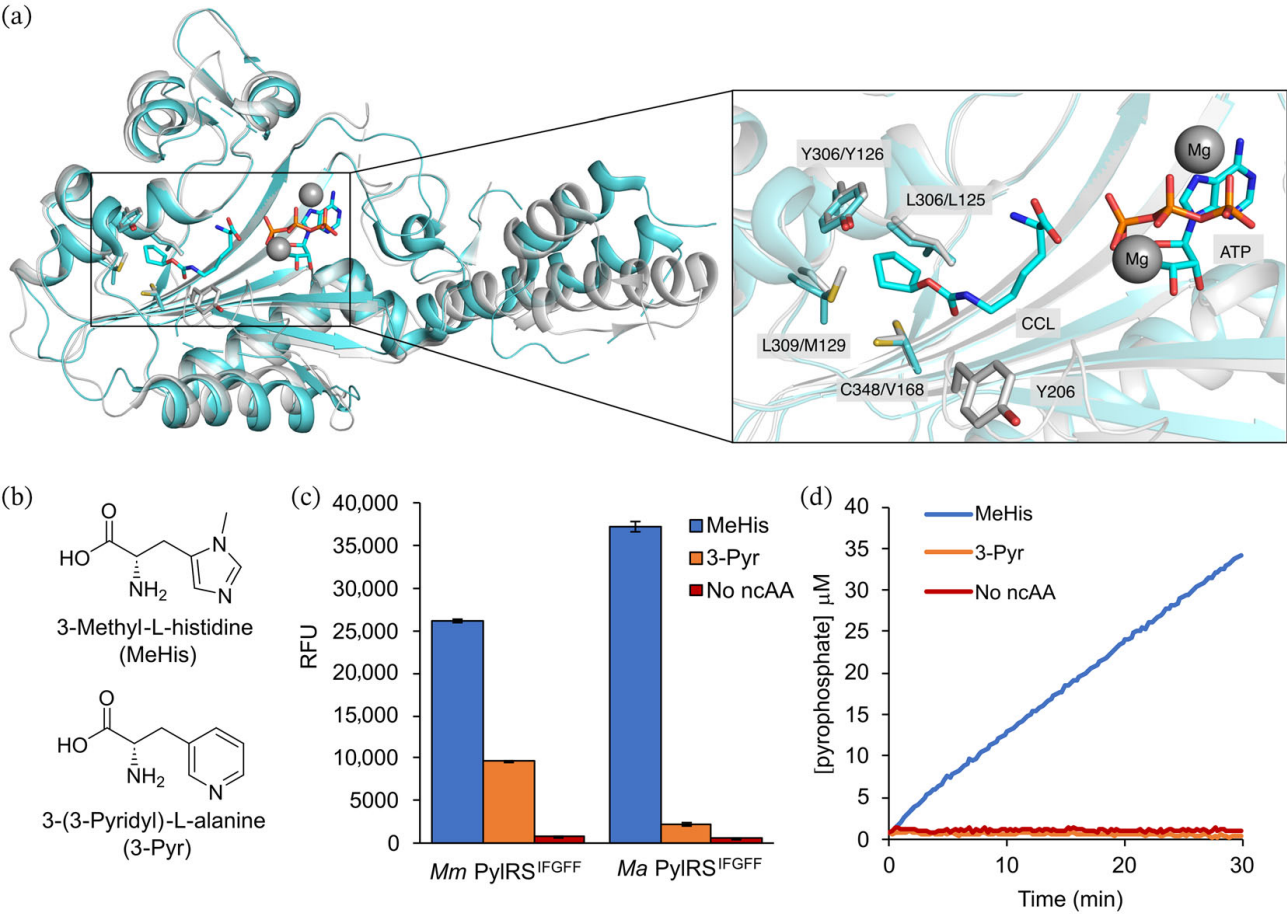
An engineered PylRS for selective MeHis incorporation. (a) An overlay of the structures of wild‐type *MmP*ylRS (gray, PDB: 2Q7G) (Krebs et al., 2007) and *Ma*PylRS (blue, PDB: 6JP2) (Gottfried‐Lee et al., 2022). The active site residues mutated in *Mm*PylRS^IFGFF^ and the analogous residues in *Ma*PylRS are shown as atom‐colored sticks with blue and gray carbons, respectively. The structure of *Mm*PylRS contains bound *N*‐ε‐[(cyclopentyloxy)carbonyl]‐L‐lysine (CCL), adenosine triphosphate (ATP), and two Mg^2+^ ions. Y384 lies on an unresolved loop in the x‐ray structure of *Mm*PylRS. (b) Structures of MeHis and 3‐Pyr. (c) Bar chart showing GFP 150 TAG expression in cultures containing MeHis (10 mM), 3‐Pyr (10 mM) or no ncAA, using either *Mm*PylRS^IFGFF^/^Pyl^tRNA_CUA_ or *Ma*PylRS^IFGFF^/^Pyl^tRNA_CUA_ pairs. Error bars represent the standard deviation of measurements made in triplicate. (d) Time course of amino acid (10 mM) adenylation with ATP (0.5 mM) catalyzed by *Ma*PylRS^IFGFF^ (2 μM). Reactions were monitored spectrophotometrically using a commercial pyrophosphate detection kit.

The structure of the C‐terminal catalytic domain of *Ma*PylRS is similar to that of *Mm*PylRS and *Mb*PylRS. In some cases, transplanting of known mutations from *Mm*PylRS to *Ma*PylRS can be used to quickly reprogram its substrate specificity (Gottfried‐Lee et al., 2022; Willis & Chin, 2018). To develop *Ma*PylRS for encoding MeHis, mutations from *Mm*PylRS^IFGFF^ (L305I Y306F L309G C348F Y384F) were mapped onto *Ma*PylRS through alignment of published structures and sequences (Figure 2a; Figure S1) (Karvan et al., 2007; Seki et al., 2020). The resulting *Ma*PylRS^IFGFF^ (L125I, Y126F, M129G, V168F, and Y206F) variant was evaluated using a GFP production assay and its activity compared to the existing *Mm* system (Figure 2b; Figure S2). Interestingly, *Ma*PylRS^IFGFF^ was not only able to suppress the UAG codon to produce full length GFP containing MeHis at position 150, but it also displayed greater efficiency than *Mm*PylRS^IFGFF^. These results demonstrate that active site transplantations from *Mm*PylRS to *Ma*PylRS can be successful even in cases where the active site has been extensively remodeled through multiple (in this instance five) mutations. Remarkably, in addition to its increased efficiency, *Ma*PylRS^IFGFF^ also displays enhanced specificity compared with *Mm*PylRS^IFGFF^ and has negligible activity toward the histidine analogue 3‐Pyr. As such, this active site transplant afforded a more efficient and selective aaRS for the incorporation of MeHis, without any requirement for further rounds of evolution to refine the structure and function.

### 2.2 *Ma*PylRS^IFGFF^ structure and aminoacylation activity

*Ma*PylRS lacks an N‐terminal tRNA binding domain that is essential for activity in *Mm*PylRS and *Mb*PylRS, and consequently it is inherently more stable to purification, more amenable to kinetic and structural characterization, and provides opportunities to produce proteins containing ncAAs using cell free expression systems (Gottfried‐Lee et al., 2022; Herring et al., 2007; Jiang & Krzycki, 2012; Seki et al., 2020). To explore the origins of *Ma*PylRS^IFGFF^ specificity, we took advantage of the favorable properties of the *Ma* system to perform MeHis and 3‐Pyr adenylation assays. Assays were performed using purified *Ma*PylRS^IFGFF^, ncAA and ATP, and monitored spectrophotometrically using a commercial pyrophosphate detection kit (EnzChek pyrophosphate assay, ThermoFisher) (Figure 2c). Consistent with our in vivo assays, *Ma*PylRS^IFGFF^ can adenylate MeHis with a rate of 0.59 ± 0.05 min^−1^ at 10 mM substrate concentration. This value is in the range of previously reported amino acid adenylation rates of PylRS variants (Guo et al., 2014; Suzuki et al., 2017; Umehara et al., 2012). In contrast, no activity is observed using 3‐Pyr as a substrate. Similarly, regioisomeric amino acids 1‐methyl‐histidine and 2‐pyridylalanine are not substrates for *Ma*PylRS^IFGFF^ (Figure S3). These data confirm that the increased selectivity observed with *Ma*PylRS^IFGFF^ arises from the specificity of amino acid adenylation rather than downstream transfer of the activated amino acid to the tRNA.

**FIGURE 3:**
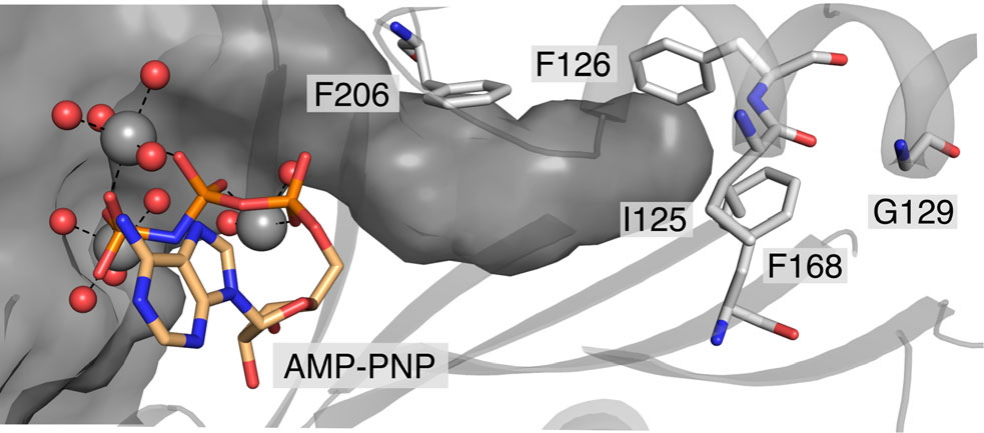
The active site of *Ma*PylRS^IFGFF^ (PDB: 8C49). Residues (atom‐colored sticks, carbons in gray) introduced into *Ma*PylRS^IFGFF^ line the active site pocket. AMP‐PNP (atom‐colored sticks, carbons in peach) and Mg^2+^ ions (gray spheres) are bound close to the amino acid binding site and overlay well with published structures of wildtype *Ma*PylRS and other variants (Gottfried‐Lee et al., 2022).

A crystal structure of *Ma*PylRS^IFGFF^ was solved (1.8 Å resolution) bound to the nonreactive ATP analogue adenylyl‐imidodiphosphate (AMP‐PNP, PDB: 8C49; Figure 3; Figure S4). The structure overlays well with the wildtype *Ma*PylRS (secondary structure rms deviation 0.56 Å, PDB: 6JP2; Figure S5). Efforts to obtain crystal structures with the amino acid substrate MeHis bound were unsuccessful, and the active site is instead occupied by an exogenous triethylene glycol from the crystallization buffer (Figure S6). As observed in published structures of *Ma*PylRS variants, AMP‐PNP is coordinated by three octahedral magnesium ions, which in turn are coordinated by water and the sidechains of residues E218, and S221 (Gottfried‐Lee et al., 2022). The flexible loop spanning residues 150–160 adopts a similar pose to that observed in AMP‐PNP/ATP bound structures of PylRS homologs. In our structure, the loop harboring residues 199–210 adopts a “closed” conformation, plausibly due to the presence of triethylene glycol positioned in the amino acid binding pocket. Efforts to solve structures containing MeHis are ongoing to understand how the mutations introduced into *Ma*PylRS give rise to the observed activity and specificity for MeHis adenylation.

### 2.3 Engineering a selective PylRS for 3‐Pyr incorporation

**FIGURE 4:**
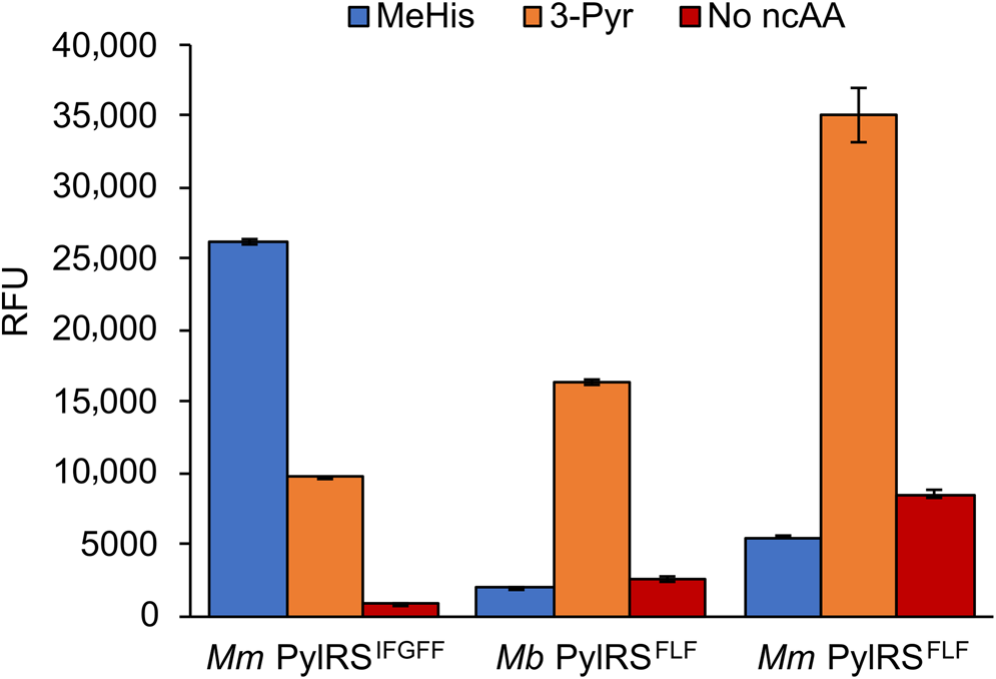
An engineered PylRS for selective 3‐Pyr incorporation. The bar chart shows GFP expression in cultures containing MeHis (10 mM, blue), 3‐Pyr (10 mM, orange) or no ncAA (red), using either *Mb*PylRS^FLF^ or *Mm*PylRS^FLF^. Error bars represent the standard deviation of measurements made in triplicate.

To take advantage of the *Ma*PylRS^IFGFF^ specificity toward MeHis, we elected to engineer selective *Mm/Mb* translation components for the incorporation of a second histidine analogue, 3‐Pyr. To this end we generated a DNA library of *Mb*PylRS variants with positions Leu270, Tyr271, Leu274, and Cys313 simultaneously randomized using NNK degenerate codons. Tyr349 was fixed as phenylalanine, a mutation previously reported to increase aminoacylation efficiency (Yanagisawa et al., 2008). This same library was previously used to select the promiscuous *Mb*PylRS^IFGFF^ variant for encoding MeHis (Xiao et al., 2014). The library was subjected to an established two‐stage selection process that links cell viability to aaRS activity and selectivity (Chin et al., 2002; Santoro et al., 2002). A single round of positive and negative selection using 3‐Pyr as a substrate converged on a single sequence containing Y271F C313L and Y349F mutations (*Mb*PylRS^FLF^). The efficiency and selectivity of the engineered synthetase was evaluated by expressing GFP 150TAG in the presence of varying 3‐Pyr and MeHis concentrations (Figure S2). The new engineered *Mb*PylRS^FLF^ variant is more efficient for 3‐Pyr incorporation than the promiscuous *Mm*PylRS^IFGFF^ variant (Figures 2b and 4). Furthermore, *Mb*PylRS^FLF^ displays a high degree of specificity for 3‐Pyr over MeHis. Transferring the Y271F C313L and Y349F mutations into *Mm*PylRS gave further improvements in UAG suppression efficiency, affording an *Mm*PylRS^FLF^ variant that is 3.5‐fold more effective for 3‐Pyr incorporation than *Mm*PylRS^IFGFF^. Successful incorporation of 3‐Pyr was confirmed by MS analysis of the intact GFP protein (Table S1).

### 2.4 Dual incorporation of MeHis and 3‐Pyr in GFP

**FIGURE 5:**
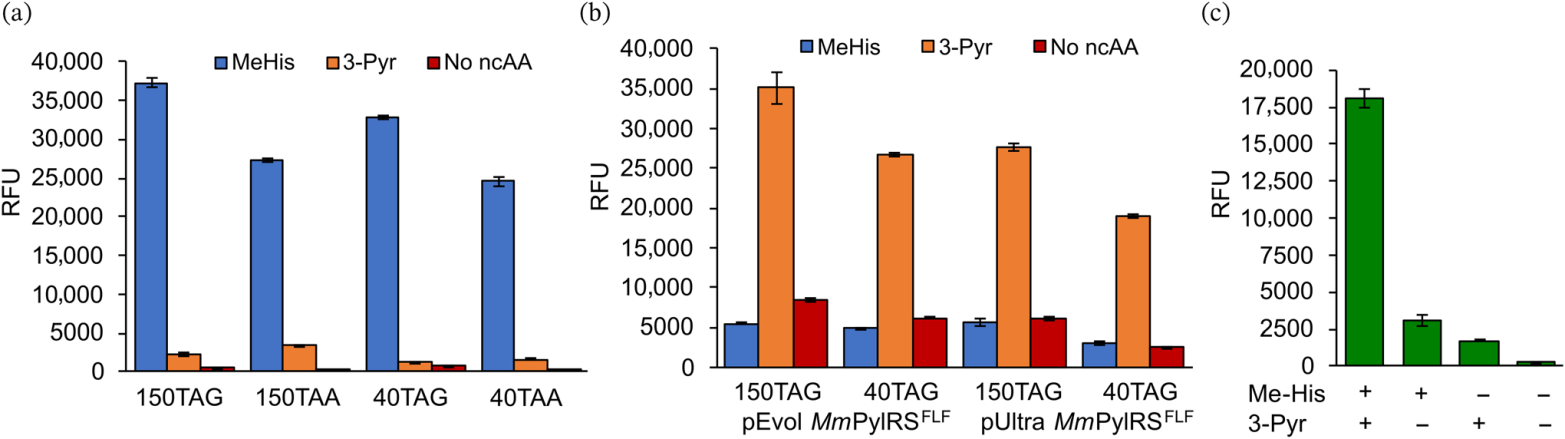
A dual incorporation system for producing proteins containing two histidine analogues. (a) Bar chart comparing the *Ma*PylRS^IFGFF^ suppression efficiency of TAA and TAG codons in GFP production assays. TAA and TAG codons were introduced at two permissive sites, Asn40 and Asn150. (b) Bar chart comparing the suppression efficiency of TAG codons by *Mm*PylRS^FLF^ */Mm* ^Pyl^tRNA_CUA_ pairs using pEVOL or pUltra expression systems. (c) Bar chart showing dual incorporation of MeHis (position 40) and 3‐Pyr (position 150) in GFP production assays using pEVOL_*Ma*PylRS^IFGFF^/*Ma* ^Pyl^tRNA_UUA_ and pUltra_*Mm*PylRS^FLF^/*Mm* ^Pyl^tRNA_CUA_ expression systems. Error bars represent the standard deviation of measurements made in triplicate.

With mutually orthogonal aaRSs for MeHis (*Ma*PylRS^IFGFF^) and 3‐Pyr (*Mm*PylRS^FLF^) incorporation in hand, attention turned to developing an expression system for dual incorporation of these histidine analogues. In addition to mutually orthogonal aaRS/tRNA pairs, site selective incorporation of two distinct ncAAs requires the use of two different nonsense codons. We elected to change the *Ma* ^Pyl^tRNA anticodon from CUA to UUA and compared the suppression efficiency of the *Ma*PylRS^IFGFF^/*Ma* ^Pyl^tRNA_CUA_ and *Ma*PylRS^IFGFF^/*Ma* ^Pyl^tRNA_UUA_ pairs in GFP production assays. In parallel, mutations were introduced into the *Ma* ^Pyl^tRNA variable loop that have been shown to prevent undesired aminoacylation of the *Ma* ^Pyl^tRNA by *Mm*PylRS (supplementary materials) (Willis & Chin, 2018). TAG and TAA mutations were introduced into the GFP gene at two permissive sites, Asn40 and Asn150. Importantly, the efficiency of UAA codon suppression was only marginally reduced compared with a UAG codon at the same position (Figure 5a). Changing the tRNA anticodon also had minimal effect on the selectivity of ncAA incorporation. We also observed that suppression of nonsense codons at position Asn150 was more efficient than at position Asn40. We next transferred the *Mm* ^Pyl^tRNA_CUA_ and *Mm*PylRS^FLF^ genes required for 3‐Pyr incorporation into a pUltra plasmid, which is mutually compatible with the pET and pEVOL plasmids harboring GFP and *Ma*PylRS^IFGFF^/*Ma* ^Pyl^tRNA_CUA_, respectively (Chatterjee et al., 2013). As expected, the resulting pUltra_ *Mm*PylRS^FLF^/*Mm* ^Pyl^tRNA_CUA_ can selectively incorporate 3‐Pyr into GFP at positions 40 and 150, with only modest reductions in UAG suppression efficiency compared with the analogous pEVOL construct (Figure 5b).

The mutually orthogonal pEVOL_*Ma*PylRS^IFGFF^/*Ma* ^Pyl^tRNA_UUA_ and pUltra_*Mm*PylRS^FLF^/*Mm* ^Pyl^tRNA_CUA_ constructs were subsequently used to produce modified GFP containing both MeHis and 3‐Pyr at positions 40 and 150, respectively. GFP production was strongly dependent on the presence of MeHis and 3‐Pyr, as evidenced by the relative fluorescence intensities of cultures grown in the presence and absence of ncAAs (Figure 5c). Successful incorporation of MeHis and 3‐Pyr was subsequently confirmed by MS analysis. Given the similar molecular masses of 3‐Pyr (166 g mol^−1^) and phenylalanine (165 g mol^−1^), we also expressed GFP in a defined media containing deuterated (d5)‐phenylalanine. MS analysis of the resulting protein further confirmed successful dual incorporation of both MeHis and 3‐Pyr (Table S1). These results demonstrate that the combination of the pUltra and pEVOL suppression systems can be extended to include mutually orthogonal PylRS/tRNA pairs for the dual incorporation of structurally similar histidine analogues.

## 3 DISCUSSION

The application of ncAAs in enzyme design and engineering is a rapidly emerging field with the potential to deliver biocatalysts for wide range of chemical transformations not found in nature (Birch‐Price et al., 2022; Drienovská & Roelfes, 2020; Lovelock et al., 2022; Zhao et al., 2020). At present, the initial activities of designed enzymes are typically low and extensive laboratory evolution is required to generate efficient catalysts. Screening of many thousands of variants containing ncAAs is greatly facilitated by the availability of efficient engineered translation components. This study highlights how the simple transplant of an engineered active site from *Mm/Mb* PylRS to a single domain homolog can give improvements in translation efficiency, even in cases where the PylRS active site has been extensively remodeled. In this regard, it is likely that further improvements can be found by exploring a wider range of pyrrolysyl homologs (Willis & Chin, 2018), or by directly engineering these systems using established selection methods. Another advantage of using single domain synthetases is their increased stability to purification, facilitating their structural characterization to aid rational re‐engineering efforts in the future. Purified synthetases can also be included in cell free expression systems to enable in vitro protein production, which can facilitate the expression of toxic or insoluble proteins. This approach has great potential to accelerate screening of large numbers of enzyme designs or libraries of engineered variants in the future (Gottfried‐Lee et al., 2022).

A next logical step in the field is to begin to design active sites containing multiple functional ncAAs. In doing so, we will greatly expand the range of catalytic mechanisms and functions accessible with designed enzymes. Here, we have taken steps toward the dual incorporation of structurally similar, functional analogues of histidine. Histidine is the most versatile amino acid in protein active sites and histidine analogues have already shown great potential in enzyme design and engineering research. Many natural enzymes contain multiple catalytically important histidines, for example, metalloenzymes that use several histidine ligands to coordinate their metal ion cofactors (Krebs et al., 2007; Lewis et al., 2011; Vaillancourt et al., 2006; Walton & Davies, 2016). We anticipate that the tools developed herein will prove useful for studying and augmenting the properties of such systems, or alternatively for creating entirely new active site mechanisms that rely on two distinct nCAAs.

## 4 MATERIALS AND METHODS

### 4.1 Materials

All chemicals and biological materials were obtained from commercial suppliers. Lysozyme, DNase I, kanamycin, and chloramphenicol were purchased from Sigma‐Aldrich, polymixin B sulfate from AlfaAesar, LB agar, LB media, 2 × YT media and arabinose from Formedium, H‐His(3‐Me)‐OH (MeHis) from Bachem, 3‐(3‐pyridyl)‐L‐alanine (3‐Pyr) from Fluorochem, 3‐(2‐pyridyl)‐L‐alanine (3‐Pyr) from AlfaAesar, 1‐methyl‐histidine from abcr, *Escherichia coli* DH10B and BL21(DE3) from Thermofisher, Q5 DNA polymerase, T4 DNA ligase and restriction enzymes from NEB and oligonucleotides were synthesized by IDT.

Defined autoinducing medium (500 mL): 25 mL aspartate (5%, pH = 7.5), 25 mL glycerol (10% w/v), 20 mL 18‐amino‐acid mix (5 g/L glutamic acid, 5 g/L aspartic acid, 5 g/L lysine.HCl, 5 g/L Arginine.HCl, 5 g/L alanine, 5 g/L proline, 5 g/L glycine, 5 g/L threonine, 5 g/L serine, 5 g/L glutamine, 5 g/L asparagine.H_2_0, 5 g/L valine, 5 g/L leucine, 5 g/L isoleucine, 5 g/L phenylalanine (or d5‐phenylalanine), 5 g/L tryptophan, 5 g/L methionine, sterile filtered), 1.25 mL arabinose (20% w/v), 10 mL lactose (10% w/v), 20 mL 25 × M salts (0.625 M NaH_2_PO_4_, 0.625 M KH_2_PO_4_, 1.25 M NH_4_Cl, 0.125 M Na_2_SO_4_), 1 mL MgSO_4_ (1 M), 0.625 mL glucose (40% w/v), 100 μL 5000 × trace metals solution (20 mM CaCl_2_.2H_2_O, 10 mM MnCl_2_.H_2_O, 10 mM ZnSO_4_.7H_2_O, 2 mM CoCl_2_.6H_2_O, 2 mM CuCl_2_, 2 mM NiCl_2_, 2 mM Na_2_MoO_4_.2H_2_O, 2 mM NaSeO_3_, 2 mM H_3_BO_3_, 50 mM FeCl_3_), sterile water to 500 mL.

### 4.2 Construction of pEVOL and pUltra suppression plasmids

pEVOL_*Mm*PylRS^IFGFF^/*Mm* ^Pyl^tRNA_CUA_ was available from a previous study (Burke et al., 2019). PylRS genes (*Ma*PylRS^IFGFF^ and *Mm*PylRS^FLF^) were optimized for *E. coli* expression and synthesized by IDT. Two copies of each gene were cloned into their respective pEVOL vectors using *BglII/SalI* and *NdeI/PstI* restriction sites. tRNAs (*Ma* ^Pyl^tRNA_CUA_, *Ma* ^Pyl^tRNA_UUA_, *Mb* ^Pyl^tRNA_CUA_) were synthesized by IDT and cloned into their respective pEVOL constructs by Gibson assembly. The *Mm*PylRS^FLF^ gene and *Mm* ^*Pyl*^ *tRNA* were also subcloned into pUltra_CNF (Addgene) using Gibson Assembly. *Ma*PylRS^IFGFF^ was subcloned into pET29 using *NdeI*/*XhoI* restriction sites.

### 4.3 GFP expression assays

Chemically competent *E. coli* BL21 (DE3) cells containing the appropriate pEVOL and/or pUltra constructs were transformed with the corresponding pET28_GFP variant. Single colonies of freshly transformed cells were cultured in 5 mL of 2 × YT medium containing 50 μg/mL kanamycin and either 25 μg/mL chloramphenicol (pEVOL) and/or 50 μg/mL spectinomycin (pUltra) for 18 h at 30°C. Expression cultures were grown in 96‐deepwell blocks (Starlabs) sealed with a breathable membrane (Greiner). Twenty microliter starter culture was used to inoculate 480 μL defined auto‐inducing medium containing appropriate antibiotics. Expression cultures were grown in the presence of ncAAs (0–10 mM) and incubated at 30°C for 48 h with shaking at 850 rpm. Cells were harvested by centrifugation (15 min, 4000 × *g*). Pelleted cells were resuspended in 400 μL of lysis buffer (PBS) containing 1 mg/mL lysozyme, 0.5 mg/mL polymyxin B sulfate and DNAse (0.01 mg/mL) and incubated for 2 h at 30°C with 850 rpm shaking. Lysates were clarified by centrifugation (30 min, 4000 × *g*) and 200 μL clarified lysates were transferred to a black 96‐well microtiter plate (Costar). Fluorescence measurements were performed using excitation 395 nm and emission 509 nm using a BMG LabTech CLARIOstar spectrophotometer.

### 4.4 *Ma*PylRS^IFGFF^ protein production and purification

For expression of *Ma*PylRS^IFGFF^, chemically competent *E. coli* BL21(DE3) were transformed with pET29_*Ma*PylRS^IFGFF^ encoding the protein with a C‐terminal 6‐His tag. A single colony of freshly transformed cells was cultured for 18 h in 10 mL LB medium containing 50 μg/mL kanamycin. Starter culture (5 mL) was used to inoculate 500 mL 2 × YT medium supplemented with 50 μg/mL kanamycin. Cultures were grown at 37°C, 200 rpm to an OD_600_ ~0.5. Protein expression was induced with the addition of IPTG to a final concentration of 0.1 mM and the culture grown for a further 20 h at 25°C.

The cells were harvested by centrifugation (12 min, 4000 × *g*), resuspended in lysis buffer (50 mM NaH_2_PO_4_, 300 mM pH = 8) and lysed by sonication (1 s on/off, 10 min, 50% amplitude). Cell lysates were cleared by centrifugation (20 min, 27,216 × *g*) and the clarified lysates subjected to affinity chromatography using Ni‐NTA Agarose (Qiagen). His‐tagged variants were eluted using 50 mM HEPES, 300 mM NaCl, pH 7.5 containing 250 mM imidazole. Purified proteins were desalted using 10DG desalting columns (Bio‐rad) with PBS pH 7.4 and analyzed by SDS‐PAGE and MS.

### 4.5 In vitro amino acid adenylation

Assays were performed using a commercial pyrophosphate detection kit (EnzChek pyrophosphate assay, ThermoFisher) according to the manufacturer's protocol. In brief, assay mix (95 μL) (containing ncAA [10 mM], 2‐amino‐6‐mercapto‐7‐methylpurine ribonucleoside [MESG, 0.2 mM], purine nucleoside phosphorylase [PNP, 0.5 U/mL], inorganic pyrophosphatase [0.01 U/mL], ATP [0.5 mM], and MgCl_2_ [1.5 mM] in 25 mM Tris–HCl with 0.05 mM sodium azide, pH 7.5) was incubated at room temperature for 15 min. The reaction was initiated with the addition of *Ma*PylRS^IFGFF^ (5 μL, 40 μM in HEPES buffer pH 7.4) and the absorbance at 360 nm was monitored over 30 min using a BMG LabTech CLARIOstar spectrophotometer.

### 4.6 Crystallization, refinement, and model building

*Ma*PylRS^IFGFF^ was crystallized by mixing 200 nL of 10 mg/mL protein in PBS pH 7.4 containing 5 mM MgCl_2_, 10 mM AMP‐PNP and 10 mM MeHis with equal volumes of precipitant. All trials were conducted by sitting‐drop vapor diffusion and incubated at 4°C. Crystallization conditions were identified using the BCS screen (Molecular Dimensions). The mother liquor contained 0.2 M magnesium chloride hexahydrate, 0.1 M tris pH 8, 25% (w/v) PEG smear (high), and 10% (w/v) glycerol. Prior to data collection crystals were cryo‐protected by the addition of 20% PEG 200 to the mother liquor and plunge cooled in liquid nitrogen. All data were collected at Diamond Light Source (Harwell, UK) using beamline i03. Data reduction was performed with Dials and the structure solved by molecular replacement using a search model derived from the structure of the wildtype *Ma*PylRS (PDB: 6JP2). Iterative rounds of model building and refinement were performed in COOT and Phenix.refine, respectively (Adams et al., 2010). Validation with MOLPROBITY and PDBREDO (Joosten et al., 2011) were incorporated into the iterative rebuild and refinement process. Data collection and refinement statistics are shown in Table S2. The coordinates and structure factors have been deposited in the Protein Data Bank under accession number 8C49.

### 4.7 PylRS library selections

#### 4.7.1 *Mb*PylRS library generation

The library was prepared by overlap extension PCR using pBK_*Mb*PylRS as a template and degenerate primer pairs (NNK codon degeneracy). The linear library fragments and the pBK vector were digested using *NdeI* and *PstI* endonucleases, gel‐purified and subsequently ligated using T4 DNA ligase.

#### 4.7.2 Positive selection

*E. coli* DH10B cells (10 × 25 μL aliquots) containing the pREP_*Mb* ^Pyl^tRNA selection plasmid encoding a chloramphenicol acetyltransferase (110TAG) were transformed with the *Mb*PylRS library. Cells were recovered in SOC (10 mL) for 1 h at which point the culture was added to non‐inducing media (1 L) containing 50 μg/mL kanamycin and 25 μg/mL tetracycline. The culture was incubated at 37°C, 200 rpm for 18 h, and then used to inoculate fresh non‐inducing media (1 L) containing antibiotics. The culture was incubated at 37°C, 200 rpm, until OD_600_ 0.8, at which point cells were plated onto LB agar plates containing 50 μg/mL kanamycin, 25 μg/mL tetracycline, 60 μg/mL chloramphenicol and 1 mM 3‐Pyr. Plates were incubated at 37°C overnight. Colonies were recovered, and the library DNA was isolated using a mini‐prep kit (Qiagen) followed by a gel extraction.

#### 4.7.3 Negative selection

*E. coli* DH10B cells (1 × 25 μL aliquots) containing the pYOBB2_*Mb* ^Pyl^tRNA selection plasmid encoding a toxic barnase gene (3TAG 45TAG) were transformed with the remaining *Mb*PylRS library members. Cells were recovered in SOC (10 mL) for 1 h and then plated onto LB agar plates containing 50 μg/mL kanamycin, 34 μg/mL chloramphenicol, and 0.2% arabinose. Plates were incubated at 37°C overnight. Colonies were recovered, and the library DNA was isolated using a mini‐prep kit (Qiagen) followed by a gel extraction.

#### 4.7.4 GFP production assay

*E. coli* DH10B cells (1 × 25 μL aliquots) containing the pALS‐sfGFP150TAG/*Mb* ^Pyl^tRNA_CUA_ plasmid encoding GFP 150TAG were transformed with the remaining library members. Cells were recovered in SOC (10 mL) for 1 h and then plated onto autoinduction agar plates containing 50 μg/mL kanamycin, 50 μg/mL tetracycline, and 1 mM 3‐Pyr. The plates were incubated at 37°C for 48 h, at which point the green colonies were picked and used to inoculate media in a 96 well plate. GFP proteins were expressed and analyzed as described above (Section 4.3).

#### 4.7.5 Validation of the most active clones

The most active clones were sequenced and successful amino acid incorporation was confirmed by MS analysis of the purified protein. For protein production and purification, glycerol stocks prepared from the overnight cultures (Section 4.7.4) were used to inoculate 5 mL non‐inducing media containing 50 μg/mL kanamycin, 34 μg/mL chloramphenicol and these were incubated at 37°C, 200 rpm for 18 h. Starter cultures were used to inoculate 50 mL defined auto‐induction medium containing appropriate antibiotics and 10 mM 3‐Pyr. Cultures were incubated at 37°C for 4 h followed by a further 44 h at 30°C. The cells were harvested by centrifugation (12 min, 4000 × *g*) and purified according to the same protocol as *Ma*PylRS^IFGFF^ (Section 4.4).

### 4.8 Dual incorporation of MeHis and 3‐Pyr in GFP

Chemically competent *E. coli* BL21 (DE3) cells containing pEVOL_*Ma*PylRS^IFGFF^/*Ma* ^Pyl^tRNA_UUA_ and pUltra_*Mm*PylRS^FLF^/*Mm* ^Pyl^tRNA_CUA_ were transformed with pET29_GFP_Asn40TAA_Asn150TAG. Single colonies of freshly transformed cells were cultured in 4 mL of 2 × YT medium containing 50 μg/mL kanamycin, 25 μg/mL chloramphenicol, and 100 μg/mL spectinomycin, for 18 h at 30°C. The overnight culture (0.5 mL) was used to inoculate 50 mL defined auto‐induction medium containing either phenylalanine or d5‐phenylalanine. Cultures were grown in the presence of 10 mM MeHis and 10 mM 3‐Pyr and were incubated at 37°C for 4 h followed by a further 44 h at 30°C. The cells were harvested by centrifugation (12 min, 4000 × *g*), resuspended in lysis buffer (50 mM NaH_2_PO_4_, 300 mM pH 8), and lysed by sonication (1 s on/off, 10 min, 50% amplitude). Cell lysates were cleared by centrifugation (20 min, 27,216 × *g*) and the clarified lysates subjected to Strep‐II purification using Strep‐Tactin Sepharose resin. GFP was eluted from the resin in lysis buffer supplemented with 5 mM desthiobiotin (Aldrich).

### 4.9 MS analysis

Purified protein samples were buffer exchanged into 0.1% acetic acid using a 10k mwco Vivaspin® (Sartorius) and diluted to a final concentration of 0.5 mg/mL. MS analysis was performed using a 1200 series Agilent LC, 5 μL injection into 5% acetonitrile (with 0.1% formic acid) and desalted inline for 1 min. Protein was eluted over 1 min using 95% acetonitrile with 5% water. The resulting multiply charged spectrum was analyzed using an Agilent QTOF 6510 and deconvoluted using Agilent MassHunter Software.

## AUTHOR CONTRIBUTIONS

**Christopher J. Taylor:** Formal analysis; investigation; methodology; visualization; writing – original draft; writing – review and editing. **Florence J. Hardy:** Investigation; writing – original draft; methodology; visualization; writing – review and editing; formal analysis. **Ashleigh J. Burke:** Investigation; writing – original draft; methodology; visualization; writing – review and editing; formal analysis. **Riley M. Bednar:** Supervision; writing – review and editing. **Ryan A. Mehl:** Supervision; writing – review and editing. **Anthony P. Green:** Conceptualization; funding acquisition; supervision; writing – review and editing; writing – original draft. **Sarah L. Lovelock:** Conceptualization; funding acquisition; writing – original draft; writing – review and editing; supervision.

## CONFLICT OF INTEREST STATEMENT

The authors declare no competing financial interest.

## Supporting information


**Data S1:** Supporting Information
