# Supplementary material for: Engineering mutually orthogonal PylRS/tRNA pairs for dual encoding of functional histidine analogues
Source: Protein Sci. 2023 May 1;32(5):e4640. doi: 10.1002/pro.4640 (PMC10127257; doi:10.1002/pro.4640)
Supplement: Supplementary file 1 — Data S1: Supporting Information [file PRO-32-e4640-s001.docx]

**Supplementary Materials**

1. **Supplementary Figures**

*Mm*PylRS CLRPMLAPN**LY**NY**L**RKLDRALPDPIKIFEIGPCYRKESDGKEHLEEFTMLNF**C**QMGSGCT 355

*Ma*PylRS ALRPMLAPN**LY**SV**M**RDLRDHTDGPVKIFEMGSCFRKESHSGMHLEEFTMLNL**V**DMGPRGD 175

*Mm*PylRS^IFGFF^ CLRPMLAPN**IF**NY**G**RKLDRALPDPIKIFEIGPCYRKESDGKEHLEEFTMLNF**F**QMGSGCT 355

*Ma*PylRS^IFGFF^ ALRPMLAPN**IF**SV**G**RDLRDHTDGPVKIFEMGSCFRKESHSGMHLEEFTMLNL**F**DMGPRGD 175

.**********. **.* .*:****:* *:****.. *********:*:**

*Mm*PylRS RE-NLESIITDFLNHLG-IDFKIVGDSCMV**Y**GDTLDVMHGDLELSSAVVGPIPLDREWGI 413

*Ma*PylRS ATEVLKNYISVVMKAAGLPDYDLVQEESDV**Y**KETIDVEINGQEVCSAAVGPHYLDAAHDV 235

*Mm*PylRS^IFGFF^ RE-NLESIITDFLNHLG-IDFKIVGDSCMV**F**GDTLDVMHGDLELSSAVVGPIPLDREWGI 413

*Ma*PylRS^IFGFF^ ATEVLKNYISVVMKAAGLPDYDLVQEESDV**F**KETIDVEINGQEVCSAAVGPHYLDAAHDV 235

*:. *: .:: * *:.:* :.. ** :*:** .. *:.**.*** ** .:

**Figure S1**. Partial sequence alignment of wild-type *Mm*PylRS, *Ma*PylRS and their respective variants that encode MeHis. *Mm*PylRS^IFGFF^ contains L305I Y306F L309G C348F and Y384F mutations; *Ma*PylRS^IFGFF^ contains L125I, Y126F, M129G, V168F and Y206F mutations.

**
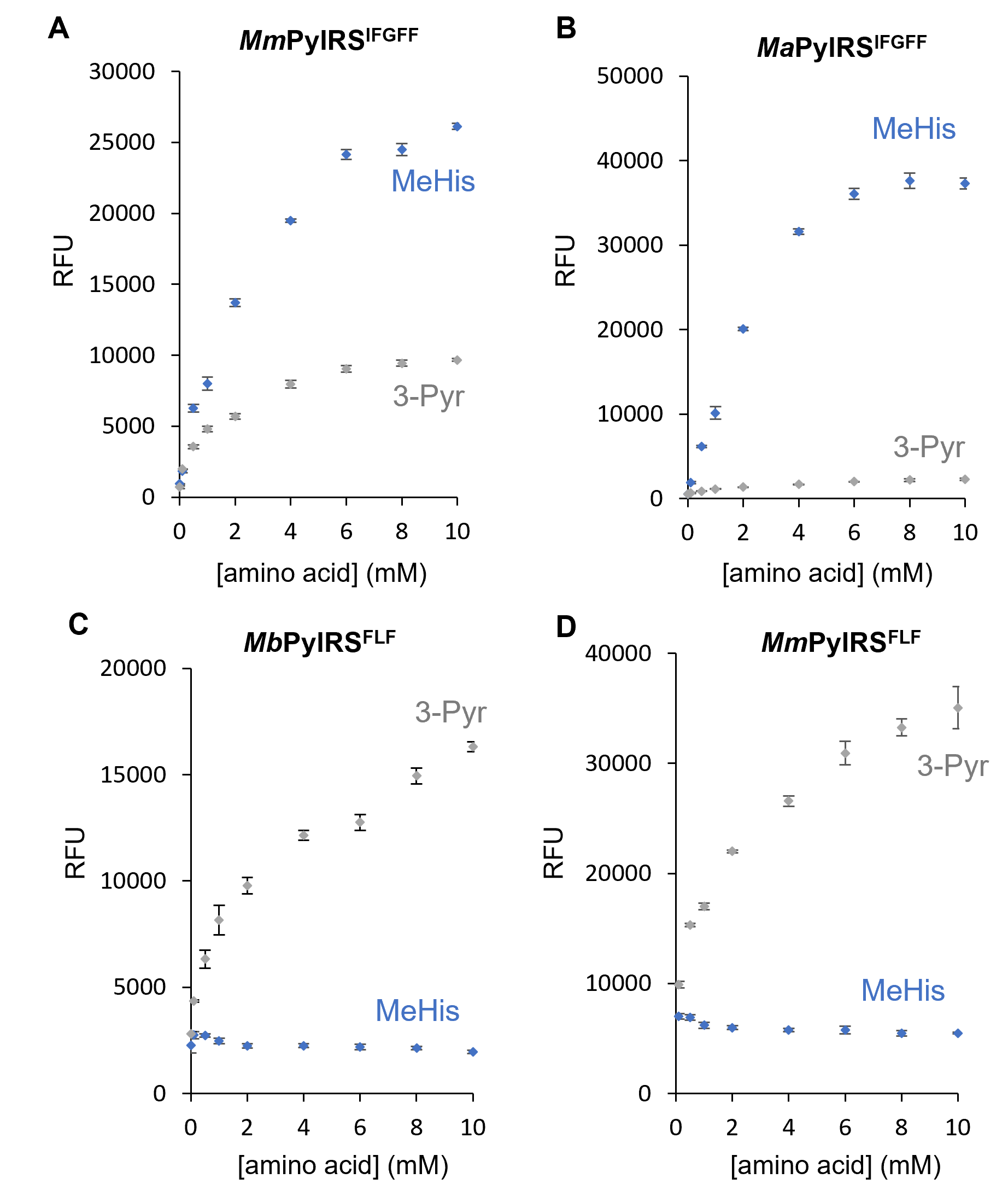
**

**Figure S2.** Expression of GFP 150TAG grown in the presence of MeHis (10 mM) or 3-Pyr (10 mM), catalyzed by **A)** *Mm*PylRS^IFGFF^**;** **B)** *Ma*PylRS^IFGFF^; **C)** *Mb*PylRS^FLF^; **D)** *Mm*PylRS^FLF^


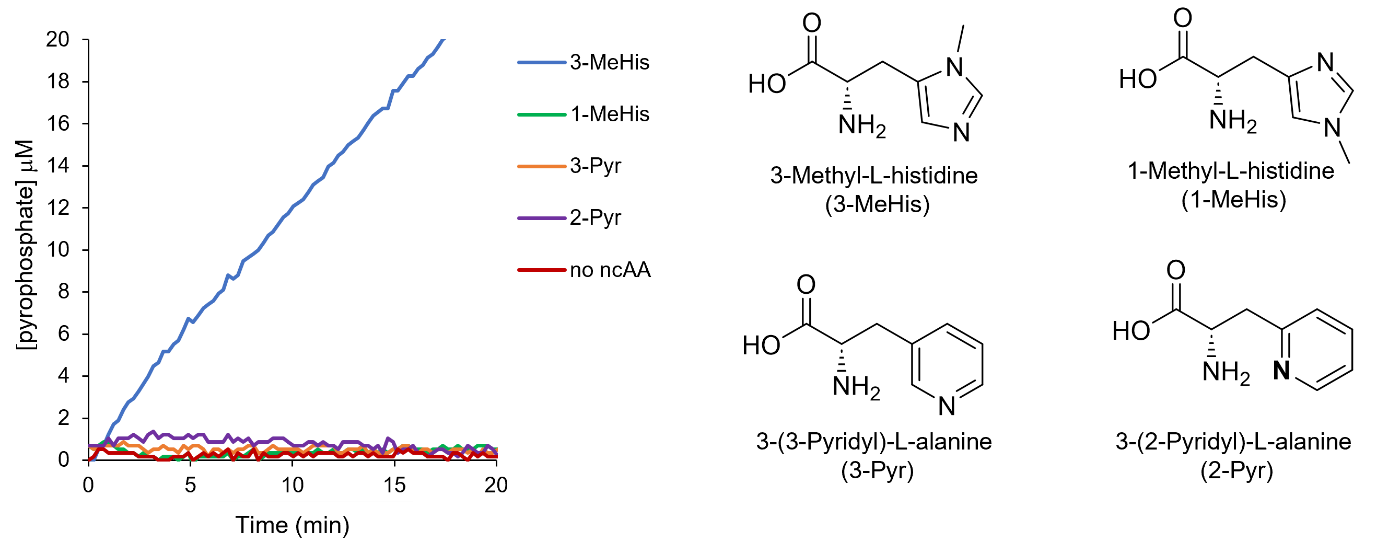


**Figure S3.** Time course of *in vitro* amino acid (10 mM) adenylation catalyzed by *Ma*PylRS^IFGFF^ (2 µM). The *Ma*PylRS^IFGFF^ variant is selective for MeHis (blue) and is not active towards 3-Pyr (orange), or regioisomeric amino acids 1-methyl-histidine (green) or 2-pyridylalanine (purple). Reactions were monitored by measuring the formation of pyrophosphate by-product.

**
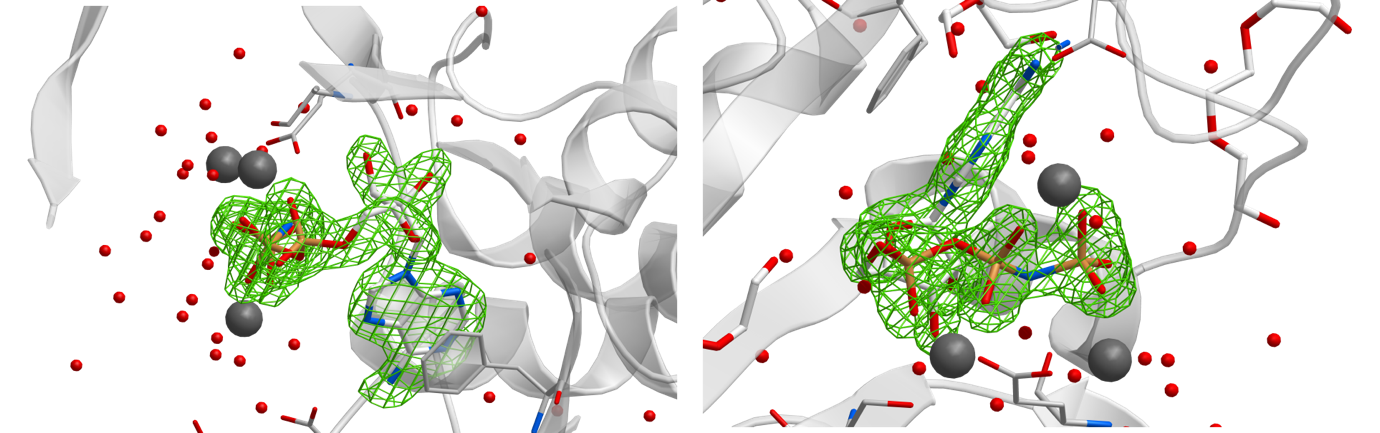
**

**Figure S4.** The active site of engineered *Ma*PylRS^IFGFF^ in complex with adenylyl-imidodiphosphate (AMP-PNP, PDB: 8C49). The Fo-Fc omit density corresponding to AMP-PNP is contoured at 3σ (green mesh). The protein is shown as a grey cartoon, water molecules as red spheres, and magnesium ions as grey spheres.

**
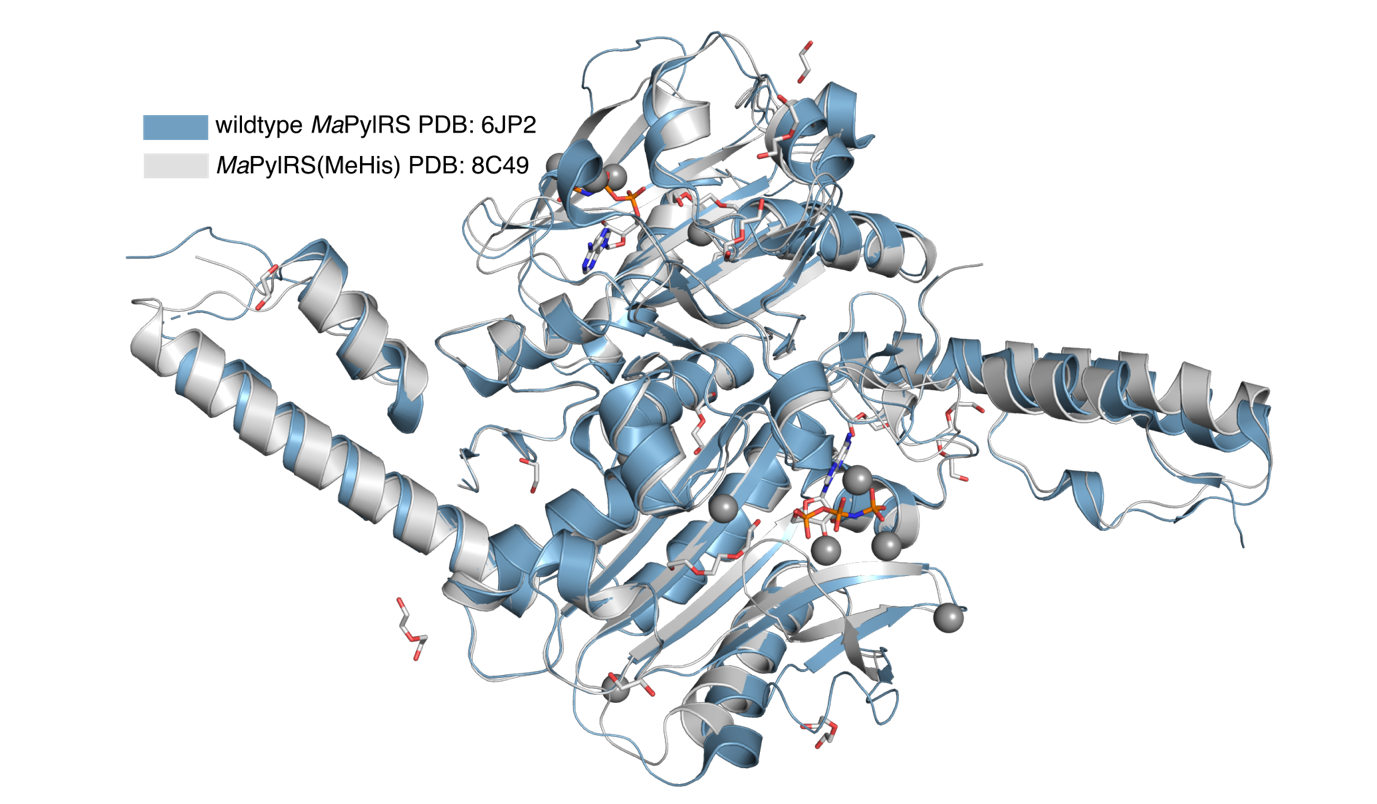
**

**Figure S5**. An overlay of wildtype *Ma*PylRS (chains A and B, PDB: 6JP2, blue cartoon) and *Ma*PylRS^IFGFF^ (grey cartoon) crystal structures. Ligands (AMP-PNP and PEG) are shown as atom-coloured sticks and magnesium ions as grey spheres.

**
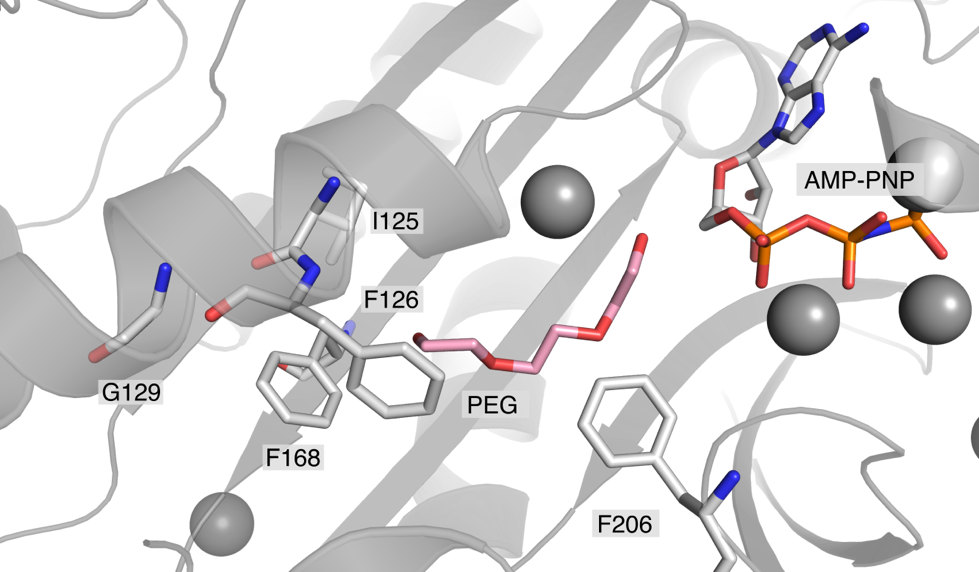
**

**Figure S6.** A PEG molecule (triethylene glycol) sits in the amino acid binding site of *Ma*PylRS^IFGFF^ in the crystal structure.

1. **Supplementary Tables**

**Table S1:** Mass spectrometry of GFP variants. See section 3 for MS data.

|  | Variant | Predicted | Observed |
| --- | --- | --- | --- |
| 1 | GFP (6His-tagged) | 27827 | 27827 |
| 2 | GFP 150 3-Pyr (6His-tagged) | 27861 | 27861 |
| 3 | GFP (strep tagged) | 28287 | 28287 |
| 4 | GFP 40 MeHis (strep tagged) | 28324 | 28323 |
| 5 | GFP 40 MeHis 150 3-Pyr (strep tagged) | 28358 | 28358 |
| 6 | GFP (d_5_-Phe) 40 MeHis (strep tagged) | 28389 | 28386 |
| 7 | GFP (d_5_-Phe) 40 MeHis 150 3-Pyr (strep tagged) | 28423 | 28419 |
| 8 | GFP (d_5_-Phe) 40 MeHis 150 d_5_-Phe (strep tagged)^a^ | 28427 | 28424 |

^a^obtained from cultures grown in d5-phenylalanine defined autoinduction media in the absence of 3-Pyr

**Table S2:** Data collection and refinement statistics

|  | *Ma*PylRS^IFGFF^ |
| --- | --- |
| PDB ID number | 8C49 |
| Wavelength (Å) | 0.9763 |
| Resolution range | 44.67 - 1.82 (1.885 - 1.82) |
| Space group | P 61 |
| Unit cell dimensions  a, b, c, (Å)  α, β, γ (°) | 59.93, 59.93, 263.23  90, 90, 120 |
| Total reflections | 1002170 (99786) |
| Unique reflections | 47516 (4638) |
| Multiplicity | 21.1 (21.5) |
| Completeness (%) | 99.74 (98.03) |
| Mean I/sigma(I) | 9.02 (1.13) |
| Wilson B-factor (Å^2^) | 23.42 |
| R-merge | 0.2091 (1.343) |
| R-meas | 0.2143 (1.375) |
| R-pim | 0.04658 (0.296) |
| CC_1/2_ | 0.997 (0.759) |
| CC* | 0.999 (0.929) |
| Reflections used in refinement | 47484 (4634) |
| Reflections used for R-free | 2358 (237) |
| R-work | 0.1836 (0.2900) |
| R-free^b^ | 0.2192 (0.3334) |
| CC (work) | 0.958 (0.820) |
| CC (free) | 0.941 (0.659) |
| RMS (bonds) | 0.004 |
| RMS (angles) | 0.73 |
| Ramachandran favoured (%) | 99.08 |
| Ramachandran allowed (%) | 0.92 |
| Ramachandran outliers (%) | 0 |
| Rotamer outliers (%) | 0.43 |
| Clashscore | 5.94 |
| Average B-factor | 29.33 |
| macromolecules | 29.14 |
| ligands | 31.13 |
| Solvent | 31.44 |

^a^Values in parentheses are for highest resolution shell. ^b^R-free was calculated using ~5% of the data separate from the rest

1. **MS data of GFP variants**

GFP (6-His-tagged)

**
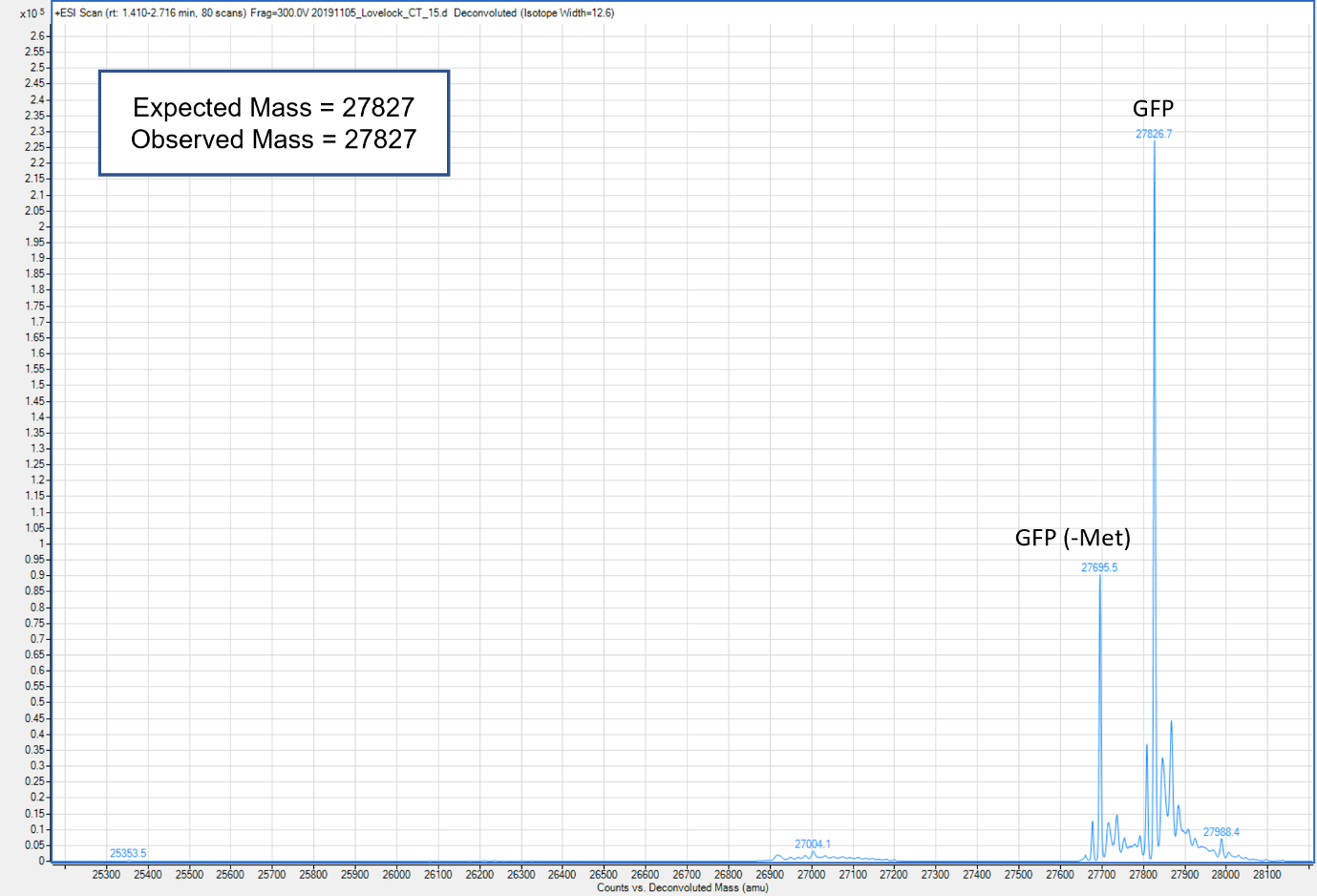
**

GFP 150 3-Pyr (6-His-tagged)


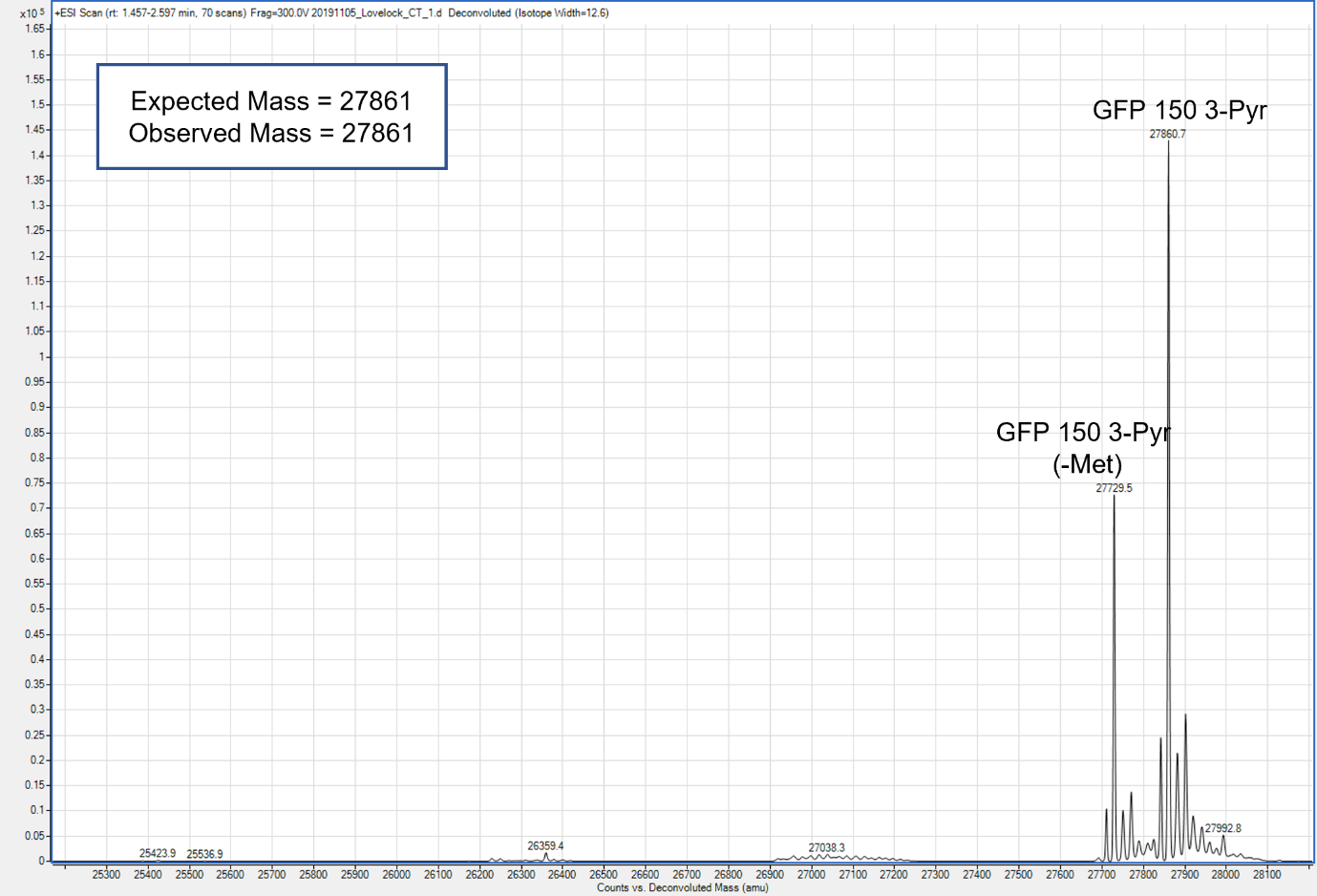


GFP (strep-tagged)


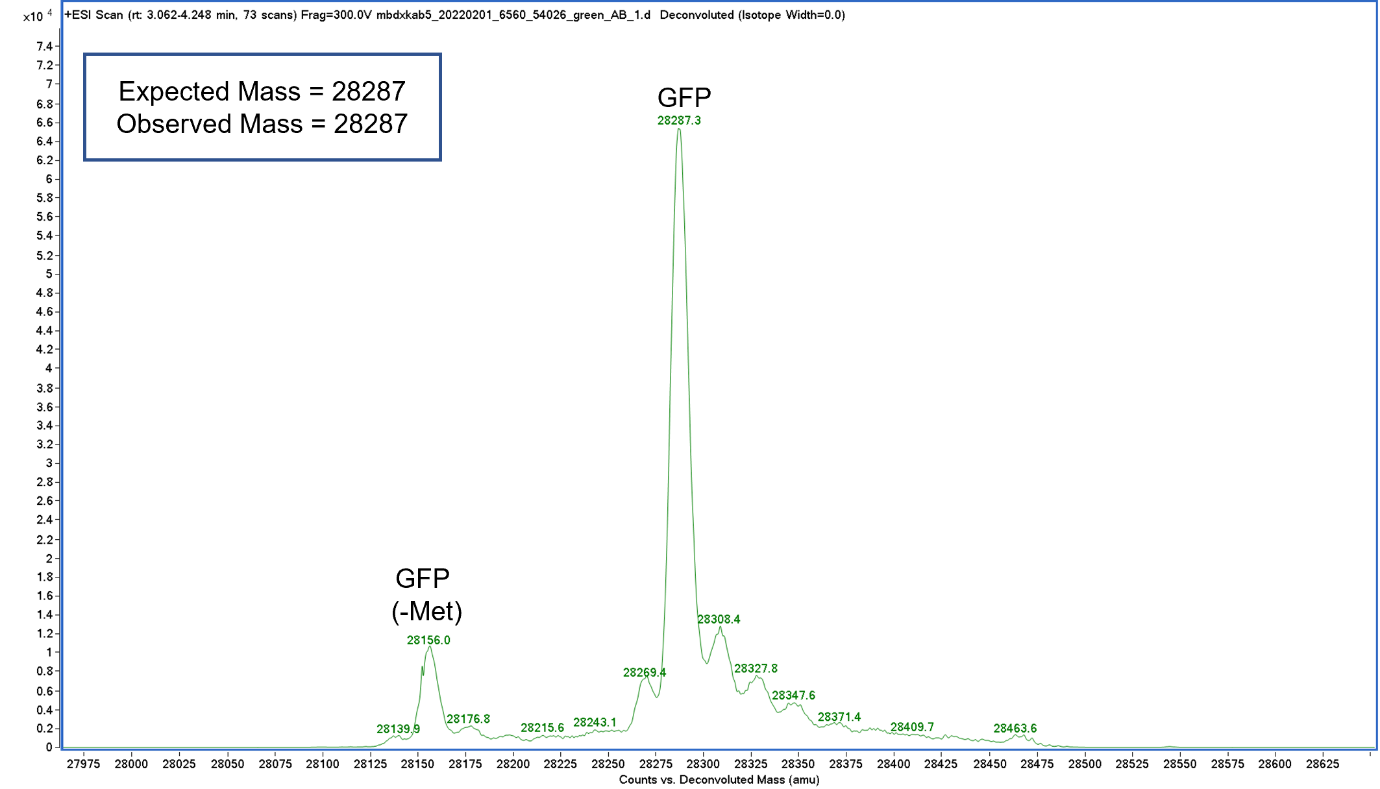


GFP 40 MeHis 150 3-Pyr (strep tagged)


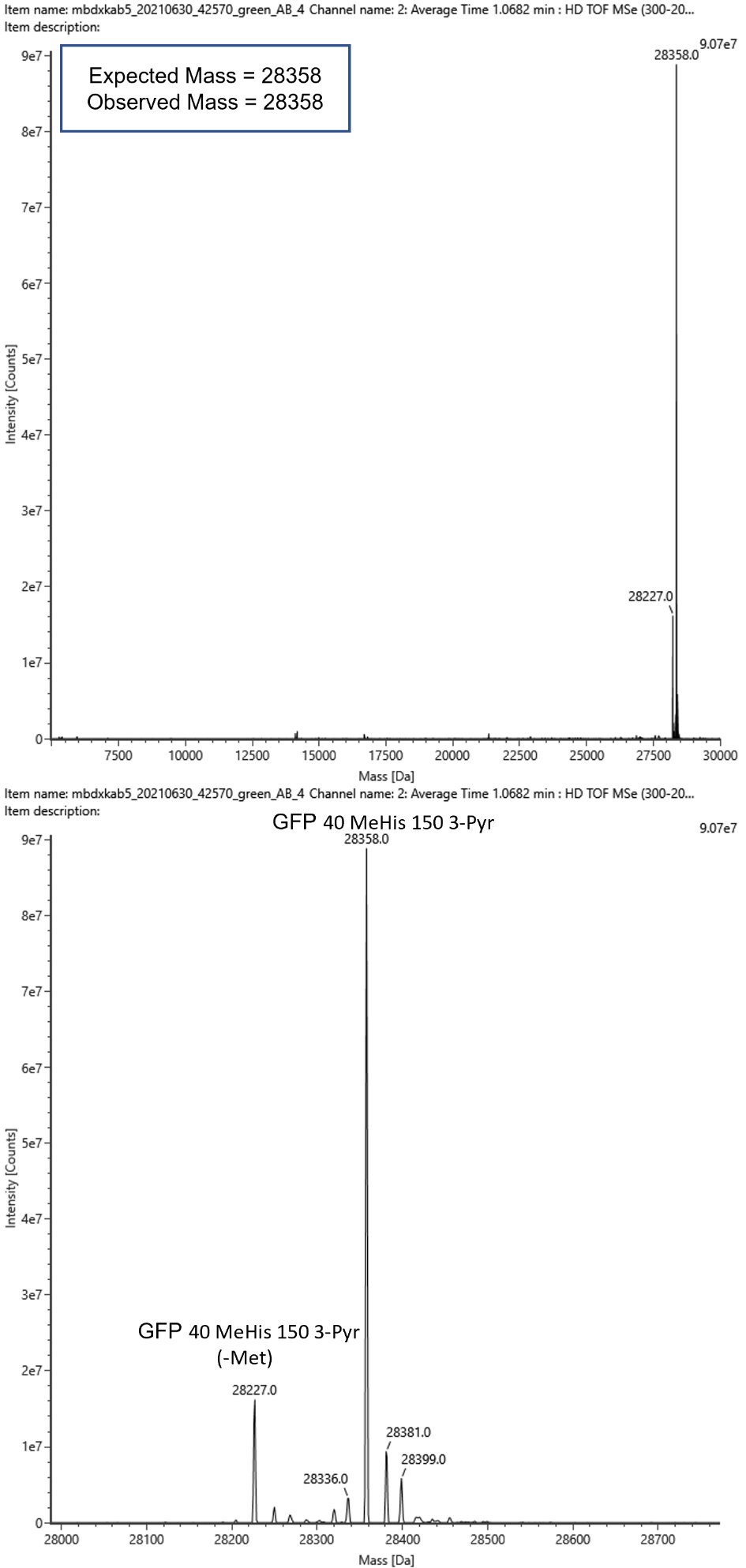


GFP (d_5_-Phe) 40 MeHis 150 3-Pyr (strep tagged)


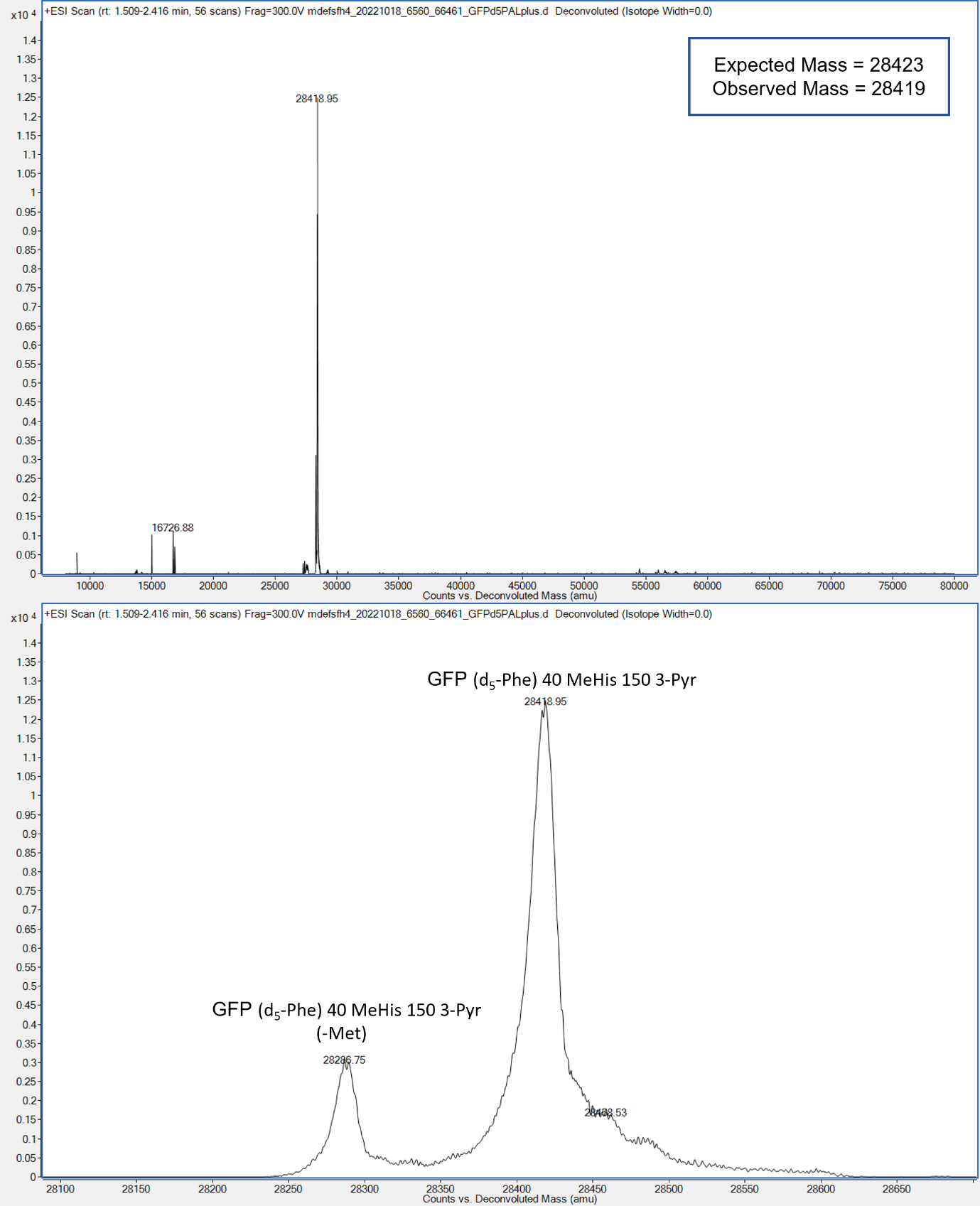


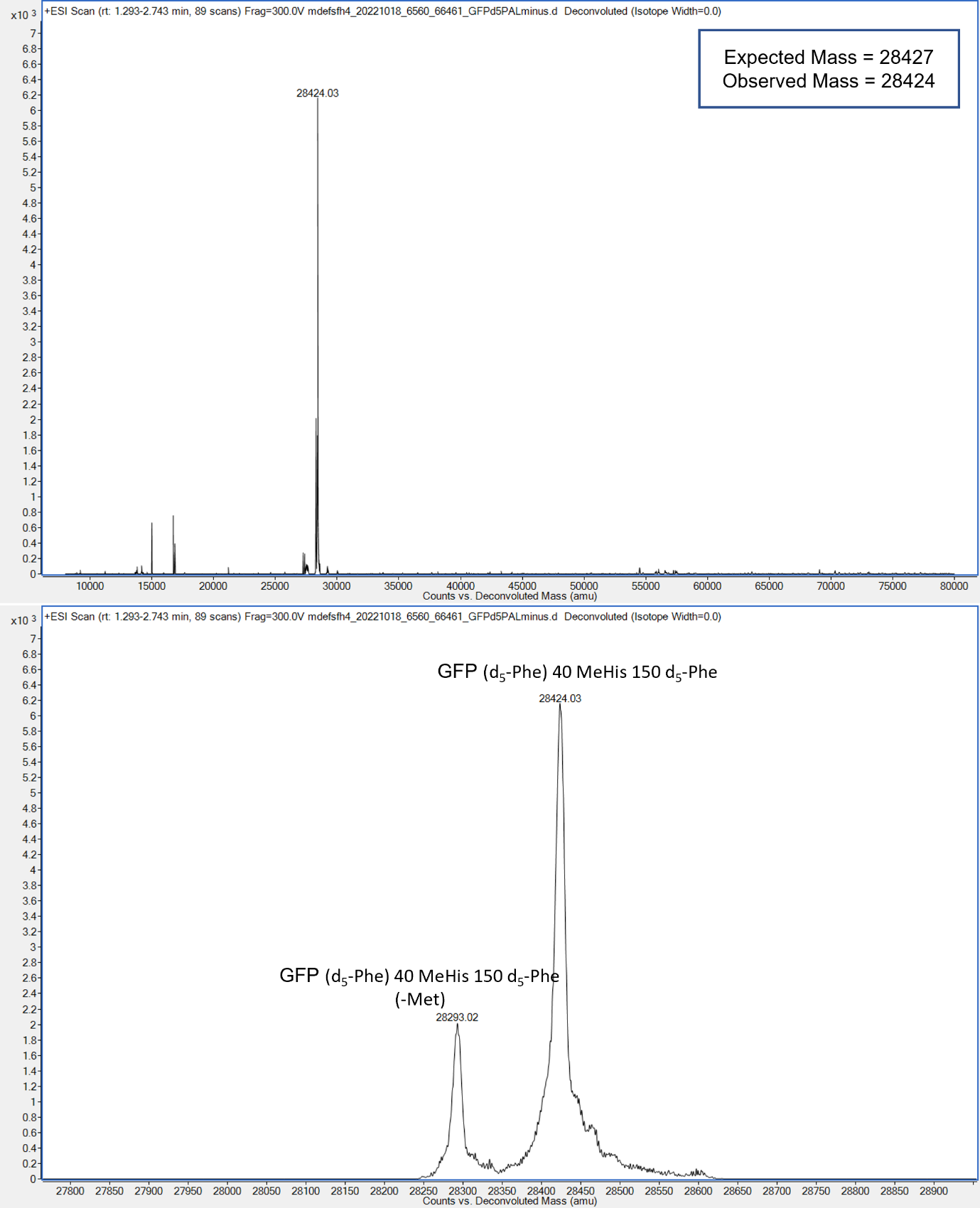
GFP (d_5_-Phe) 40 MeHis 150 d_5_-Phe (strep tagged)

1. **DNA and protein sequences**

***Mm*PylRS^IFGFF^**

atggacaaaaaaccgctgaataccctgatctctgctactggtctgtggatgagtcgtaccggaaccattcataaaatcaaacaccacgaggttagccgttcgaaaatctatattgagatggcgtgtggcgatcatctggttgtgaacaatagccgctcttctcgtacagcacgtgcactgcgtcaccacaaatatcgtaaaacctgtaaacgttgccgtgtgtccgatgaggatctgaacaaattcctgacaaaagccaatgaggaccaaacaagcgtgaaagtgaaagtcgttagcgctcctacccgtactaaaaaagcaatgccgaaatccgttgctcgtgcccctaaaccactggaaaacactgaagcagcacaggcacagccgtctggaagcaaattctctccggccattcctgtttctacccaggagtccgtttctgttccagcaagtgtgagcaccagcattagcagtattagcaccggtgccaccgctagcgccctggttaaaggcaataccaatccgattacaagcatgtctgccccggttcaagcatcagctccagcactgacaaaatcccaaaccgatcgtctggaggttctgctgaatccgaaagacgaaatcagcctgaattccggcaaaccgtttcgtgaactggagagcgaactgctgtcacgtcgtaaaaaagacctgcaacaaatctatgccgaagaacgtgagaactatctggggaaactggaacgtgaaatcacccgctttttcgtggatcgtggctttctggagatcaaatccccgattctgattcctctggagtatatcgagcgtatgggcatcgacaatgataccgaactgagcaaacaaattttccgtgtggataaaaacttctgtctgcgccctatgctggcaccaaatatctttaactatggtcgcaaactggaccgtgccctgcctgatcctatcaaaatcttcgagatcggcccgtgttatcgtaaagagtccgacggtaaagaacatctggaggagtttaccatgctgaactttttccaaatgggttcaggttgtactcgtgagaacctggaaagcatcatcaccgattttctgaaccacctgggcattgacttcaaaattgtgggcgacagctgtatggtgtttggcgacaccctggatgtcatgcacggcgacctggaactgtctagtgccgttgttggaccaattccgctggaccgtgagtggggtatcgacaaaccgtggatcggagcaggattcggtctggaacgcctgctgaaagtgaaacacgacttcaaaaacatcaaacgtgccgcccgttctgaatcgtattataacgggatctctacgaacctg

MDKKPLNTLISATGLWMSRTGTIHKIKHHEVSRSKIYIEMACGDHLVVNNSRSSRTARALRHHKYRKTCKRCRVSDEDLNKFLTKANEDQTSVKVKVVSAPTRTKKAMPKSVARAPKPLENTEAAQAQPSGSKFSPAIPVSTQESVSVPASVSTSISSISTGATASALVKGNTNPITSMSAPVQASAPALTKSQTDRLEVLLNPKDEISLNSGKPFRELESELLSRRKKDLQQIYAEERENYLGKLEREITRFFVDRGFLEIKSPILIPLEYIERMGIDNDTELSKQIFRVDKNFCLRPMLAPN**IF**NY**G**RKLDRALPDPIKIFEIGPCYRKESDGKEHLEEFTMLNF**F**QMGSGCTRENLESIITDFLNHLGIDFKIVGDSCMV**F**GDTLDVMHGDLELSSAVVGPIPLDREWGIDKPWIGAGFGLERLLKVKHDFKNIKRAARSESYYNGISTNL

***Ma*PylRS^IFGFF^** atgacggtgaagtacacggacgcacaaatccaacgtctgcgcgaatatgggaatggaacttatgagcagaaggtattcgaggatttagcatcacgtgatgcagcattctcaaaagaaatgtctgttgcaagcactgataatgaaaaaaaaattaaggggatgatcgctaacccttctcgtcacggattaacccagttaatgaatgacattgcagatgcacttgtagctgagggctttatcgaagtgcgtaccccgatcttcattagtaaggacgcgcttgcccgtatgacaattacagaagacaagcccctgttcaagcaggtgttctggatcgacgaaaaacgtgctcttcgccccatgttggcccccaatatcttctctgttggccgtgacttgcgcgaccacacggatggacctgtgaaaatctttgaaatggggtcctgtttccgcaaagagtcacatagtggcatgcatttagaagaatttacaatgttaaacttatttgatatgggacctcgtggggacgcgaccgaggtacttaagaactacatttcagtggtgatgaaagctgccggcctgcctgactatgaccttgtgcaagaagaaagtgatgtcttcaaggagactattgacgtcgaaattaacggtcaggaggtatgctcggcggccgtgggccctcattatcttgatgcagcgcatgatgtccatgaaccttggtcaggagccgggttcgggttggaacgtctgttgacaatccgcgagaaatattcgacggtgaaaaagggcggagcgtccatcagctatcttaacggggcgaaaattaat

MTVKYTDAQIQRLREYGNGTYEQKVFEDLASRDAAFSKEMSVASTDNEKKIKGMIANPSRHGLTQLMNDIADALVAEGFIEVRTPIFISKDALARMTITEDKPLFKQVFWIDEKRALRPMLAPN**IF**SV**G**RDLRDHTDGPVKIFEMGSCFRKESHSGMHLEEFTMLNL**F**DMGPRGDATEVLKNYISVVMKAAGLPDYDLVQEESDV**F**KETIDVEINGQEVCSAAVGPHYLDAAHDVHEPWSGAGFGLERLLTIREKYSTVKKGGASISYLNGAKIN

***Mb*PylRS^FLF^**

atggataaaaaaccgctggatgtgctgattagcgcgaccggcctgtggatgagccgtaccggcaccctgcataaaatcaaacatcatgaagtgagccgcagcaaaatctatattgaaatggcgtgcggcgatcatctggtggtgaacaacagccgtagctgccgtaccgcgcgtgcgtttcgtcatcataaataccgcaaaacctgcaaacgttgccgtgtgagcgatgaagatatcaacaactttctgacccgtagcaccgaaagcaaaaacagcgtgaaagtgcgtgtggtgagcgcgccgaaagtgaaaaaagcgatgccgaaaagcgtgagccgtgcgccgaaaccgctggaaaatagcgtgagcgcgaaagcgagcaccaacaccagccgtagcgttccgagcccggcgaaaagcaccccgaacagcagcgttccggcgtctgcgccggcaccgagcctgacccgcagccagctggatcgtgtggaagcgctgctgtctccggaagataaaattagcctgaacatggcgaaaccgtttcgtgaactggaaccggaactggtgacccgtcgtaaaaacgattttcagcgcctgtataccaacgatcgtgaagattatctgggcaaactggaacgtgatatcaccaaattttttgtggatcgcggctttctggaaattaaaagcccgattctgattccggcggaatatgtggaacgtatgggcattaacaacgacaccgaactgagcaaacaaattttccgcgtggataaaaacctgtgcctgcgtccgatgctggccccgaccttgtttaattatttgcgtaaactggatcgtattctgccgggtccgatcaaaatttttgaagtgggcccgtgctatcgcaaagaaagcgatggcaaagaacacctggaagaattcaccatggttaactttttgcagatgggcagcggctgcacccgtgaaaacctggaagcgctgatcaaagaattcctggattatctggaaatcgacttcgaaattgtgggcgatagctgcatggtgtttggcgataccctggatattatgcatggcgatctggaactgagcagcgcggtggtgggtccggttagcctggatcgtgaatggggcattgataaaccgtggattggcgcgggttttggcctggaacgtctgctgaaagtgatgcatggcttcaaaaacattaaacgtgcgagccgtagcgaaagctactataacggcattagcacgaacctg

MDKKPLDVLISATGLWMSRTGTLHKIKHHEVSRSKIYIEMACGDHLVVNNSRSCRTARAFRHHKYRKTCKRCRVSDEDINNFLTRSTESKNSVKVRVVSAPKVKKAMPKSVSRAPKPLENSVSAKASTNTSRSVPSPAKSTPNSSVPASAPAPSLTRSQLDRVEALLSPEDKISLNMAKPFRELEPELVTRRKNDFQRLYTNDREDYLGKLERDITKFFVDRGFLEIKSPILIPAEYVERMGINNDTELSKQIFRVDKNLCLRPMLAPTL**F**NYLRKLDRILPGPIKIFEVGPCYRKESDGKEHLEEFTMVNF**L**QMGSGCTRENLEALIKEFLDYLEIDFEIVGDSCMV**F**GDTLDIMHGDLELSSAVVGPVSLDREWGIDKPWIGAGFGLERLLKVMHGFKNIKRASRSESYYNGISTNL

***Mm*PylRS^FLF^**

atggacaaaaaaccgctgaataccctgatctctgctactggtctgtggatgagtcgtaccggaaccattcataaaatcaaacaccacgaggttagccgttcgaaaatctatattgagatggcgtgtggcgatcatctggttgtgaacaatagccgctcttctcgtacagcacgtgcactgcgtcaccacaaatatcgtaaaacctgtaaacgttgccgtgtgtccgatgaggatctgaacaaattcctgacaaaagccaatgaggaccaaacaagcgtgaaagtgaaagtcgttagcgctcctacccgtactaaaaaagcaatgccgaaatccgttgctcgtgcccctaaaccactggaaaacactgaagcagcacaggcacagccgtctggaagcaaattctctccggccattcctgtttctacccaggagtccgtttctgttccagcaagtgtgagcaccagcattagcagtattagcaccggtgccaccgctagcgccctggttaaaggcaataccaatccgattacaagcatgtctgccccggttcaagcatcagctccagcactgacaaaatcccaaaccgatcgtctggaggttctgctgaatccgaaagacgaaatcagcctgaattccggcaaaccgtttcgtgaactggagagcgaactgctgtcacgtcgtaaaaaagacctgcaacaaatctatgccgaagaacgtgagaactatctggggaaactggaacgtgaaatcacccgctttttcgtggatcgtggctttctggagatcaaatccccgattctgattcctctggagtatatcgagcgtatgggcatcgacaatgataccgaactgagcaaacaaattttccgtgtggataaaaacttctgtctgcgccctatgctggcaccaaatctgtttaactatctgcgcaaactggaccgtgccctgcctgatcctatcaaaatcttcgagatcggcccgtgttatcgtaaagagtccgacggtaaagaacatctggaggagtttaccatgctgaactttttgcaaatgggttcaggttgtactcgtgagaacctggaaagcatcatcaccgattttctgaaccacctgggcattgacttcaaaattgtgggcgacagctgtatggtgtttggcgacaccctggatgtcatgcacggcgacctggaactgtctagtgccgttgttggaccaattccgctggaccgtgagtggggtatcgacaaaccgtggatcggagcaggattcggtctggaacgcctgctgaaagtgaaacacgacttcaaaaacatcaaacgtgccgcccgttctgaatcgtattataacgggatctctacgaacctg

MDKKPLNTLISATGLWMSRTGTIHKIKHHEVSRSKIYIEMACGDHLVVNNSRSSRTARALRHHKYRKTCKRCRVSDEDLNKFLTKANEDQTSVKVKVVSAPTRTKKAMPKSVARAPKPLENTEAAQAQPSGSKFSPAIPVSTQESVSVPASVSTSISSISTGATASALVKGNTNPITSMSAPVQASAPALTKSQTDRLEVLLNPKDEISLNSGKPFRELESELLSRRKKDLQQIYAEERENYLGKLEREITRFFVDRGFLEIKSPILIPLEYIERMGIDNDTELSKQIFRVDKNFCLRPMLAPNL**F**NYLRKLDRALPDPIKIFEIGPCYRKESDGKEHLEEFTMLNF**L**QMGSGCTRENLESIITDFLNHLGIDFKIVGDSCMV**F**GDTLDVMHGDLELSSAVVGPIPLDREWGIDKPWIGAGFGLERLLKVKHDFKNIKRAARSESYYNGISTNL

***Mb*^pyl^tRNA_CUA_**

tggcggaaaccccgggaatctaacccggctgaacggatttagagtccattcgatctacatgatcaggttccc

***Ma*^pyl^tRNA_CUA_**

gggggacggtccggcgaccagcgggtct**cta**aaacctagccagcggggttcgacgccccggtctctcgcca

***Mm*^pyl^tRNA_CUA_**

ggaaacctgatcatgtagatcgaatggact**cta**aatccgttcagccgggttagattcccggggtttccgcca

***Ma*^Pyl^tRNA_UUA:_** The UUA anti-codon and mutations to the variable loop that prevent undesired aminoacylation of the *Ma*^Pyl^tRNA by *Mm*PylRS are shown in bold.

gggggacggtccggcgaccagcgggtct**tta**aaacctagc**atag**cggggttcgac**a**ccccggtctctcgcca
